# Fumonisin B_1_ Inhibits Cell Proliferation and Decreases Barrier Function of Swine Umbilical Vein Endothelial Cells

**DOI:** 10.3390/toxins13120863

**Published:** 2021-12-03

**Authors:** Qing Li, Qiaoling Yuan, Tianjie Wang, Yang Zhan, Lingchen Yang, Ying Fan, Hongyu Lei, Jianming Su

**Affiliations:** Provincial Key Laboratory of Protein Engineering in Animal Vaccines, College of Veterinary Medicine, Hunan Agricultural University, Changsha 410128, China; qingli@stu.hunau.edu.cn (Q.L.); yql0501@126.com (Q.Y.); wtj19980420@163.com (T.W.); yangzhan@hunau.edu.cn (Y.Z.); lcyang@hunau.edu.cn (L.Y.); fany1025@126.com (Y.F.)

**Keywords:** Fumonisin B_1_, porcine umbilical vein endothelial cells, cell proliferation, barrier function

## Abstract

The fumonisins are a group of common mycotoxins found around the world that mainly contaminate maize. As environmental toxins, they pose a threat to human and animal health. Fumonisin B_1_ (FB_1_) is the most widely distributed and the most toxic. FB_1_ can cause pulmonary edema in pigs. However, the current toxicity mechanism of fumonisins is still in the exploratory stage, which may be related to sphingolipid metabolism. Our study is designed to investigate the effect of FB_1_ on the cell proliferation and barrier function of swine umbilical vein endothelial cells (SUVECs). We show that FB_1_ can inhibit the cell viability of SUVECs. FB_1_ prevents cells from entering the S phase from the G1 phase by regulating the expression of the cell cycle-related genes cyclin B1, cyclin D1, cyclin E1, Cdc25c, and the cyclin-dependent kinase-4 (CDK-4). This results in an inhibition of cell proliferation. In addition, FB_1_ can also change the cell morphology, increase paracellular permeability, destroy tight junctions and the cytoskeleton, and reduce the expression of tight junction-related genes claudin 1, occludin, and ZO-1. This indicates that FB_1_ can cause cell barrier dysfunction of SUVECs and promote the weakening or even destruction of the connections between endothelial cells. In turn, this leads to increased blood vessel permeability and promotes exudation. Our findings suggest that FB_1_ induces toxicity in SUVECs by affecting cell proliferation and disrupting the barrier function.

## 1. Introduction

Fumonisins are the primary mycotoxins produced by Fusarium spp. [1]. These molecules are diesters of propane-1,2,3-tricarboxylic acid with similar long-chain aminopolyol backbones [2]. FB_1_ is considered toxicologically the most hazardous as it constitutes the majority of naturally occurring fumonisins in food and feed [3]. Fumonisins can cause human esophageal cancer, equine leukoencephalomalacia, hepatotoxicity in mammals, atherosclerosis in primates, and other diseases or conditions [4,5,6,7]. Pulmonary edema was demonstrated to consistently develop 4–7 days after starting to feed FB_1_ to pigs [8,9,10]. The accumulation of membrane material was found in pulmonary intravascular macrophages of pigs with pulmonary edema where the primary site was identified as pulmonary capillary endothelial cells [11]. In addition, edema fluid appeared around blood vessels when fumonisins caused pulmonary edema in pigs [12].

Cell proliferation is a fundamental characteristic of living organisms, which requires the accumulation of intracellular biomass. Accordingly, cell metabolism is essential for a replicative cell division [13]. And toxins may cause the termination of the cell cycle by disrupting metabolism. Cells, the cornerstone of life, reproduce themselves by progressing through a series of events that is referred to as the cell cycle. This is a complex process common to cell growth and proliferation, which involves the regulation of DNA damage repair and steps like those that take place in cancer [14]. The steps are regulated by several families of molecules, namely cyclin, cyclin-dependent kinase (CDKs) and substrate proteins, CDK inhibitor (CKI), and tumor suppressor gene p53 [15,16]. These families of molecules and genes achieve the remarkable tasks of cell cycle transitions and checkpoint.

Endothelial cells connect to each other to form a cell layer that covers the surface of blood vessels to form a barrier. The special connections between adjacent cells ensure that these cellular barriers are not only stable and tight, but remain permeable. Endothelial cells utilize two types of specialized junctional complexes to mediate cell-cell interactions: tight junctions (TJs) and adhesion junctions (AJs) [17]. Tight junctions are intermingled with adhesion junctions along the cleft. Together with the cytoskeleton, they form an endothelial barrier to the endothelial cells [18]. Tight junctions consist mainly of transmembrane proteins (occludin, claudin and JAM) and cytoplasmic proteins (ZO-1, ZO-2, ZO-3) [19]. The ability of cells to maintain and change morphology, and to generate, transmit, and respond to mechanical signals depends on the cytoskeleton, a network structure of filamentous polymers and regulatory proteins in eukaryotic cells. These physiological processes are mediated by three types of cytoskeletal filament: actin, microtubules, and intermediate filaments [20]. When the dynamic equilibrium between these filaments is broken, it may lead to the rearrangement of the cytoskeleton. This affects the normal structure and function of the cell, which thus destroys cell junctions [21]. Many reports support the idea that tight junctions, adhesion junctions, and the cytoskeleton are interconnected, and that they constitute the cell barrier. In this way, the changes in tight junctions and adhesion junctions are related to the structure of the cytoskeleton.

The structural integrity of vascular endothelial cells is the basis of their normal function. We speculate that the main reasons of fumonisin induced pulmonary edema is closely related to the changes of vascular endothelial cells. However, there are very few reports on the mechanism of FB_1_-induced pulmonary edema in pigs [11]. Swine umbilical vein endothelial cells (SUVECs) are commonly used as an in vitro model for studying features of vascular disease. In the present study, we focused on investigating the effects of Fumonisin B_1_ on proliferation and barrier function of swine umbilical vein endothelial cells.

## 2. Results

### 2.1. FB_1_ Reduces Cell Viability and Causes Cell Damage

**Figure 1:**
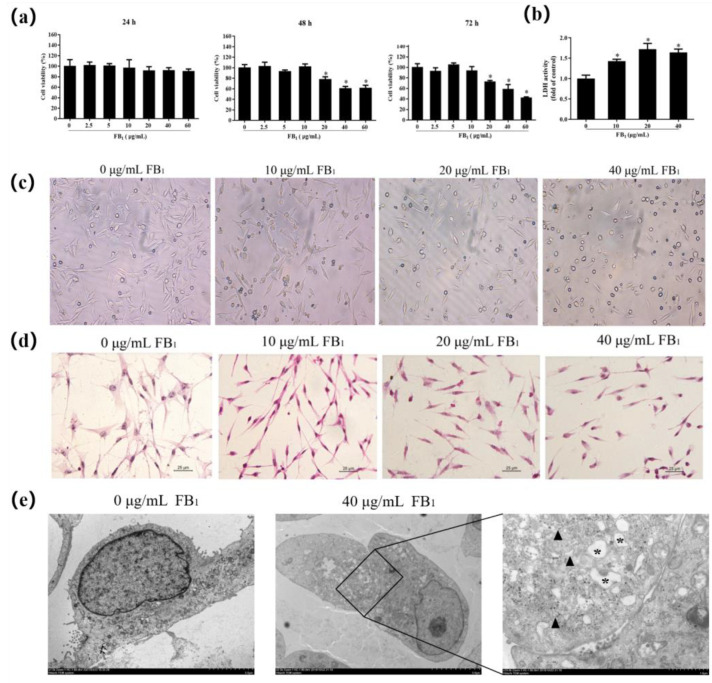
FB_1_ reduces cell viability and changes cell morphology. (**a**) Cell viability was measured after FB_1_ exposure for 24, 48, and 72 h; (**b**) Effect of different concentrations of FB_1_ for 48 h on LDH activity in the supernatant of SUVECs; (**c**) The cell morphology of FB_1_-treated groups under light microscopy (×200); (**d**) HE staining of cell morphology (×400); (**e**) Representative TEM images showing the cell damage to SUVECs; the endoplasmic reticulum (black arrowheads) and mitochondrial swelling (black asterisk) were observed. The data were expressed as mean ± SD. * *p* < 0.05.

MTT assays were performed after exposure of FB_1_ for 24, 48, and 72 h to evaluate the viability of SUVECs (Figure 1a). Exposure to 20, 40, and 60 μg/mL FB_1_ for 48 h and 72 h significantly reduced cell viability (*p* < 0.05). FB_1_ concentrations of 10, 20, and 40 μg/mL were subsequently investigated for 48 h. The LDH content in the FB_1_ treatment group increased significantly in a dose-dependent manner compared with the control group (Figure 1b, *p* < 0.05), and the increase was concentration dependent. This indicates that FB_1_ can reduce cell viability and increase the activity of LDH in the cell supernatant of SUVECs.

After FB_1_ treatment for 48 h, the experimental set of cells shriveled, and the intercellular junctions decreased significantly. The cells became round and clearly shriveled, and many cells died (Figure 1c). This was particularly apparent at a concentration of 40 μg/mL FB_1_. The connections between cells at 40 μg/mL FB_1_ were faintly visible (Figure 1d). FB_1_ therefore affects the cell morphology of SUVECs and causes cell shrinkage. Next, we evaluated whether exposure of FB_1_ could affect cell damage in SUVECs using TEM. The dilatation of the endoplasmic reticulum and the observed mitochondrial swelling were significantly increased in the FB_1_-treated groups (Figure 1e). Collectively, these data suggest that exposure of FB_1_ causes damage to SUVECs.

### 2.2. FB_1_ Inhibits Proliferation of SUVECs by Blocking the Cell Cycle

**Figure 2:**
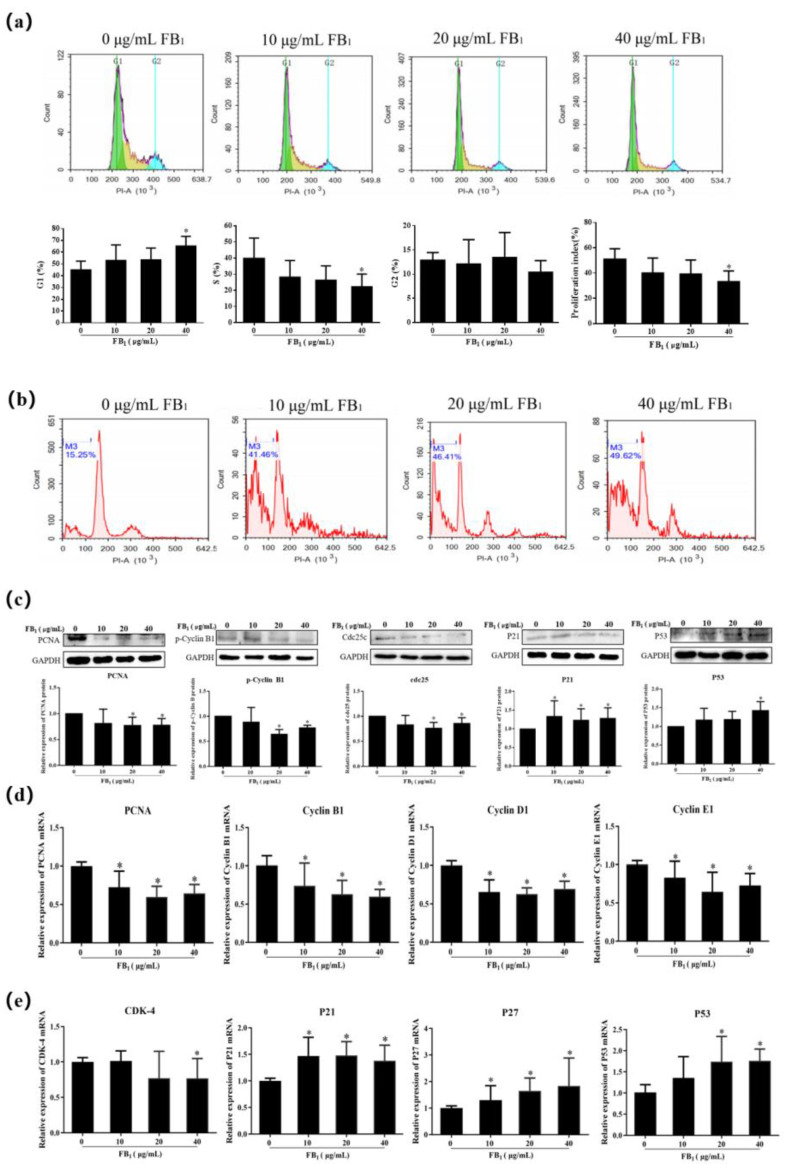
FB_1_ blocks the cell cycle from the G1 phase to the S phase, thereby affecting cell proliferation. (**a**) Flow detection diagram of cell cycle of SUVECs treated with FB_1_ for 48 h; (**b**) Flow detection diagram of late apoptosis of SUVECs treated with FB_1_ for 48 h; (**c**) The effect of FB_1_ on the protein expression of PCNA, cyclin B1, Cdc25c, p21, and p53; (**d**) The effect of FB_1_ on the mRNA expression of PCNA, cyclin B1, cyclin D1, and cyclin E1; (**e**) The effect of FB_1_ on the mRNA expression of CDK-4, p21, p27, and p53. The data were expressed as mean ± SD. * *p* < 0.05.

After 48 h of FB_1_ exposure, the proportion of cells in the G1 phase in the 40 μg/mL group was significantly increased. The proportion of cells in the S phase was downregulated, while the proportion of cells in the G2 phase did not change (Figure 2a). The cell proliferation index decreased as the concentration of FB_1_ increased in the FB_1_ treatment group: in the 40 μg/mL FB_1_ treatment group, the cell proliferation index decreased from (51.06 ± 8.232)% to (33.34 ± 8.38)%. The proportion of late apoptosis was significantly increased (Figure 2b). FB_1_ therefore blocks the progression of the cell cycle from the G1 phase to the S phase.

The previously described experiments demonstrated that FB_1_ inhibited cell proliferation. Next, the expression of PCNA was detected. The expression of PCNA was significantly downregulated (Figure 2c,d, *p* < 0.05). We detected the related genes cyclin D1, cyclin E1, and CDK-4 that regulate the G1 and S phases of the cell cycle to corroborate the effect of FB_1_ on the progression of the cell cycle from the G1 phase to S phase (Figure 2d,e). FB_1_ clearly reduced the relative expression level of cyclin D1 and cyclin E1 mRNA. In addition, CDK-4 mRNA expression was decreased at 40 μg/mL of FB_1_. This shows that FB_1_ inhibits the relevant regulatory genes in the G1 and S phases, thereby blocking the progression of cell proliferation from the G1 phase to the S phase. Next, we tested the expression of cyclin B1 and Cdc25c, the regulators of the cell M phase (Figure 2c,d). FB_1_ inhibited the expression of cyclin B1 and Cdc25c. Therefore, FB_1_ inhibits the expression of Cdc25c and cyclin B1 related to the regulation of the M phase.

The cyclin-dependent kinase inhibitors p21 and p27 were examined to further investigate the role of FB_1_ on the cell cycle. The expression of p21 was upregulated (Figure 2c,e), and FB_1_ increased the mRNA level of p27 in a dose-dependent manner (Figure 2e). p53 is a tumor suppressor gene and is involved in the cell cycle process. As expected, FB_1_ also increased the expression of p53 (Figure 2c,e). This result indicated that FB_1_ promotes cyclin-dependent kinase inhibitor expression, thereby inhibiting cell proliferation.

### 2.3. FB_1_ Destroys the Tight Junctions of SUVECs

**Figure 3:**
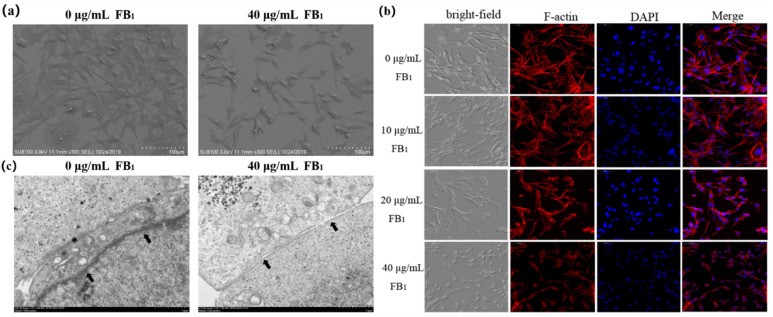
FB_1_ destroys the tight junctions of SUVECs. (**a**) Representative SEM images showing the ultrastructure of SUVECs (×300); (**b**) Representative phalloidin images showing microfilament skeleton of SUVECs (×200); (**c**) Representative TEM images showing the cell connection of SUVECs (×10,000); the destruction of tight junctions (black arrowheads) was observed. The data was expressed as mean ± SD.

In the FB_1_ treatment group, the cells clearly shrank, exhibited a small and elliptical morphology, the filopodia of the cells were significantly reduced or even broken, and the cell surface became smooth (Figure 3a). FB_1_ can therefore change the basic morphology of cells, causing changes in the structure of cell membranes and the junction. Cells were treated with phalloidin to further explore the functional effects of FB_1_ on the barrier function, and the cell microfilament skeleton was observed (Figure 3b). The microfilaments were arranged in an orderly manner in the control group, while an abnormal cytoskeleton system was found in the FB_1_-treated group. In the 10 μg/mL group, the arrangement of the microfilament was disordered although there was no significant reduction in the number of cells. In high concentration groups, the cell microfilament skeleton was disorganized. In the 40 μg/mL-treated group, it was significantly reduced. FB_1_ can therefore cause abnormalities in the cytoskeleton system of SUVECs, leading to disordered arrangement and distribution of microfilament skeletons. After FB_1_ treatment, the tight junctions between the cells were destroyed (Figure 3c). These observations indicated that FB_1_ can break the tight junctions of cells.

### 2.4. FB_1_ Increases the Cell Paracellular Permeability of SUVECs

**Figure 4:**
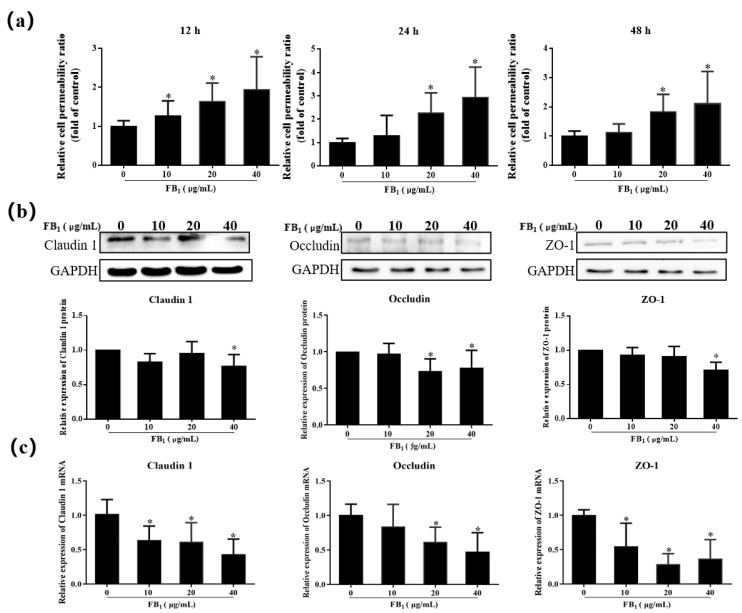
FB_1_ increases the cell membrane permeability of SUVECs. (**a**) Histogram of relative cell paracellular permeability in FB_1_-treated cells for 12, 24, and 48 h; (**b**) The effect of FB_1_ on the protein expression of claudin 1, occludin, and ZO-1; (**c**) The effect of FB_1_ on the mRNA expression of claudin 1, occludin, and ZO-1. The data were expressed as mean ± SD. * *p* < 0.05.

SUVECs were exposed to FB_1_ for 12, 24, and 48 h. The paracellular permeability increased in the FB_1_ treatment group compared with the control group (Figure 4a), which indicated that FB_1_ can increase the paracellular permeability of SUVECs.

Therefore, the relative expression of the cell tight junction-related genes claudin1, occludin, and ZO-1 were further tested to further explore the action of FB_1_ on paracellular permeability (Figure 4b,c). When the concentration of FB_1_ was 40 μg/mL, the protein expression of claudin 1 and ZO-1 decreased significantly, and mRNA expression had a notable decrease in the FB_1_ treatment group (*p* < 0.05). In addition, the expression of occludin in the 20–40 μg/mL group decreased significantly (*p* < 0.05). FB_1_ treatment for 48 h can therefore inhibit the expression of the tight junction proteins claudin 1, occludin, and ZO-1 in SUVECs.

## 3. Discussion

FB_1_ is a highly toxic low molecular weight fusarium mycotoxin. [22]. It has species-specific toxicity that threatens the health of humans and animals [23]. Pulmonary edema is a unique pathological change due to the action of FB_1_ in pigs [24]. In this experiment, we found that FB_1_ caused cell proliferation inhibition and barrier dysfunction in SUVECs, which provides new insights into the toxic mechanism of FB_1_ in porcine pulmonary edema.

This study explored the effects of different concentrations of FB_1_ on SUVECs cells. The inhibitory effect of FB_1_ on the cell activity of SUVECs was time- and dose-dependent. Previously, Zhao and coworkers found that cell viability decreased for human umbilical vein endothelial cells when they were treated with 50 μM/L FB_1_ for 24 h and 48 h [25]. In our previous study, FB_1_ also had an inhibitory effect on the cell viability of porcine hip artery endothelial cells when the concentration reached 50 μg/mL [26]. Morphological observations can further prove that FB_1_ inhibits cell viability. LDH is a cytoplasmic enzyme that exists in all cells. When the cell membrane is damaged, LDH is rapidly released into the supernatant, which can impact cell proliferation, apoptosis, and other biological characteristics [27,28]. In our experiment, the LDH activity in the cell culture supernatant increased. We observed the dilatation of endoplasmic reticulum and mitochondrial swelling using TEM. These findings indicate that FB_1_ caused damage to the SUVECs.

We speculate that FB_1_ can affect cell viability due to the cell cycle being blocked, which results in cells being unable to replicate, divide, and proliferate normally. The cell cycle can be divided into the pre-DNA replication phase (G1 phase), DNA replication phase (S phase), late DNA replication phase (G2 phase), mitotic phase (M phase), and stationary phase (G0 phase) [29,30]. Cell cycle detection by flow cytometry showed that G1 phase cells accounted for a notable increase in the proportion of total cells, while the number of S phase cells decreased. Therefore, FB_1_ may interrupt proliferation by blocking cells in the transition from the G1 phase to the S phase. In addition, many reports in the literature support this. Bouhet et al. [31] found that FB_1_ blocked the proliferation of intestinal porcine epithelial cell line-1 (IPEC-1) in the G0/G1 phase in a concentration-dependent manner. Our results are consistent with these findings. They demonstrate that FB_1_ can inhibit cell proliferation, mainly by inhibiting the progression of the cell cycle from the G0/G1 phase to the S phase. PCNA plays a critical role in the initiation of DNA replication and replication-associated processes and is a good indicator of the state of cell proliferation [32,33]. In our study, the mRNA and protein expression levels of PCNA were significantly down-regulated, which proves that FB_1_ inhibited cell proliferation.

The cell cycle is a complex process, which includes cyclin interaction [34]. Cyclin D is expressed in the early G1 phase. It simultaneously forms a cell cycle kinase complex with CDK4 and CDK6 and becomes the promoter of the cell cycle [35]. In this study, the mRNA expression of Cyclin D1 and CDK4 was significantly down-regulated in the FB_1_ treatment group. The expression of the cyclin E gene, which regulates the transition of the cell cycle from the G1 to S phase, was also significantly decreased. Cdc25c, an important cell division regulatory protein, is one of the key factors that controls the progression of the cell cycle into the M phase [36,37], cyclin B also participates in regulating M phase progression. We found that the mRNA and protein expression levels of Cdc25c and cyclin B1 decreased significantly, which was consistent with the previous flow cytometry results. This further shows that the arrest of the cell cycle by FB_1_ occurs during the progression from the G1 phase to the S phase. Ciacci-zanella and coworkers [38] determined that FB_1_ blocked cell-cycle progression in CV-1 cells, and the expression of cyclin E, Cdc2, CDK2, CDK3, and CDK4 in cells after FB_1_ exposure was significantly inhibited. Our experimental results are consistent with these findings. CDK inhibitors P21 and P27 can promote cell cycle arrest in response to a variety of stimuli and prevent the replication of damaged DNA [39,40,41]. p53 can up-regulate the expression of p21 and, subsequently, p21 protein binds to cyclins E/Cdk2 and D/Cdk4, which leads to cell cycle G1 arrest [42]. p53 can also bind to other p53 target genes, such as Cdc25 [43] to block the cell cycle in the G2/M phase. Western-blot and qRT-PCR test results confirmed that p21, p27, and p53 were significantly up-regulated. The study also found that the decrease of CDK-4 and cyclin caused by FB_1_ may be related to the expression of p21 and p27. It remains to be explored whether there are other ways to down-regulate its expression such as post-transcriptional control of non-coding RNA regulation or methylation and ubiquitination-mediated protein degradation.

The cytoskeleton refers to the protein fiber network structure in eukaryotic cells. It helps to maintain cell shape and bear external forces. When cells are attacked, the function of the cytoskeleton is destroyed, and microfilaments and microtubules play an important role in this process [44]. The inhibition of the proliferation of vascular smooth muscle cells (VCMC) is associated with a rapid rearrangement of cytoskeleton [45]. Baltierra-Uribe and coworkers [46] found that when endothelial cells are damaged by mycobacterial infection, the actin filaments are lost, and multiple actin focal points appear in the infected cells. Qin and colleagues [47] also reported that when tight junctions are injured, the cytoskeleton arrangement is disordered and even broken. In our current study, we found that FB_1_ can cause damage to vascular endothelial cells and affect the cytoskeleton, which leads to the destruction of the tight junctions, further induce the enhancement of vascular permeability and the exudation of intravascular substances.

Vascular endothelial cells and their junctional complexes are the main basis for the structure and function of the vascular barrier. The connections between endothelial cells, which are closely related to vascular permeability, include tight junctions and adhesion junctions [48]. Tight junctions play a vital role in maintaining the morphology of endothelial cells, the integrity of the barrier function, cell polarity, and cell-to-cell transport [49]. Adhesive junctions adhere to adjacent cells by connecting their actin cytoskeleton, while tight junctions seal the paracellular space between overlapping endothelial cells [50]. Damage to cells’ tight junctions can lead to cells where the lateral gap expands. We demonstrated that FB_1_ expanded the paracellular space of SUVECs, which results in increased paracellular permeability. We explored whether FB_1_ could damage the tight junction of SUVECs based on these results. Claudins, occludin, and ZO-1 are commonly occurring proteins that play a very important role in maintaining cell morphology and forming barriers to prevent the invasion of foreign pathogenic factors [51,52]. In our study, FB_1_ inhibited gene and protein expression. TEM showed that the connections between cells in the FB_1_ treatment group were damaged. This phenomenon may arise as FB_1_ changes the connection between cells and destroys tight junction structure between cells. Similarly, studies have shown that a long exposure of FB_1_ can significantly change the barrier function [31], and cause the downregulation of claudin gene expression in intestinal cells [53]. It has also been reported that 10–50 μg/mL FB_1_ disrupts the barrier function of IPEC-J2 after 48 h, which results in the destruction of cell tight junctions, and decline of the expression of claudin 1, occludin, and ZO-1 [54]. Zhang and colleagues [55] showed that variation in HUVEC cell permeability down-regulates the expression of ZO-1 and changes the ultrastructure of tight junctions, which leads to a significant increase in the intercellular space. In summary, FB_1_ can destroy the tight junction of vascular endothelial cells.

## 4. Conclusions

**Figure 5:**
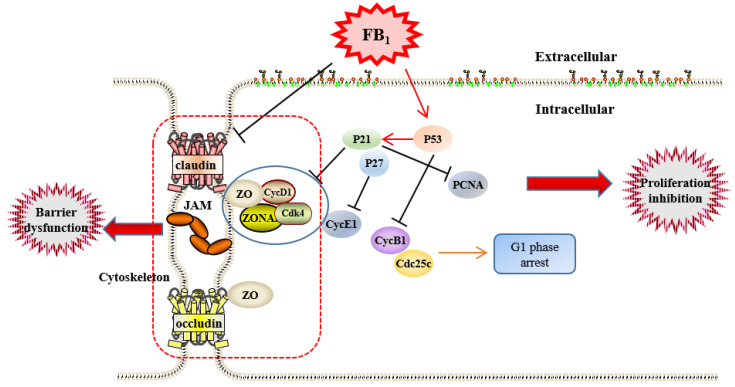
Fumonisin B_1_ inhibits cell proliferation and decreases barrier function of swine umbilical vein endothelial cells.

Our study with SUVECs cultured in vitro reveals that the vascular toxicity of FB_1_ is related to triggers SUVECs damage, inhibits cell proliferation by arresting the cell cycle in G1 phase, destroys tight junctions and cytoskeleton (Figure 5). These findings provide insight into our understanding of the toxicity mechanism of FB_1_, and may facilitate its potential application for the treatment of swine pulmonary edema.

## 5. Materials and Methods

### 5.1. Cell and Cell Culture

SUVECs were bought from Shanghai Zishi Bio-Technology Co., Ltd (Shanghai, China). The cell morphology of SUVEC is polygonal or long fusiform with relatively uniform size. The cells were cultured in Dulbecco’s modified eagle medium (DMEM, Thermo, Fisher Scientific Co., Ltd. Shanghai, China) which contained 10% fetal calf serum (FBS, Gibco) and 1% penicillin-streptomycin (Procell Life Science &Technology Co., Ltd. Beijing, China) at 37 °C in a cell incubator containing 5% carbon dioxide.

### 5.2. Cell Viability Assay

MTT (3-(4,5-dimethylthiazol-2-yl)-2,5-diphenyltetrazolium bromide) assay was used to measure the cell viability of SUVECs. FB_1_ (≥98%, Sigma-Aldrich, St. Louis, MO, USA) was dissolved in dimethyl sulphoxide (DMSO) to make a concentration of 20 mg/mL. Exponentially growing cells were seeded into 96-well culture plates. Different concentrations of FB_1_ (0, 2.5, 5,10, 20, 40, 60 μg/mL) were added and samples were incubated for 24, 48, and 72 h. Cells were cultured for a further 4 h after replacing the FB_1_ with 110 μL thiazolyl blue (MTT) (CWBIO, Beijing, China). Finally, the MTT solution was discarded and 150 μL DMSO (CWBIO) was added. The absorbance was measured at 490 nm using an enzyme-labeled method in a spectrophotometer. The activity of each group of cells was calculated according to the formula: cell viability (%) = (A test − A blank)/(A control − A blank) × 100%.

### 5.3. LDH Cytotoxicity Assay

SUVECs were grown to a cell density of about 1 × 10^6^ in the 6-well plate. At this time, the medium was replaced with 2 mL FB_1_ solution (0, 10, 20, 40 μg/mL). The cell culture supernatant was collected from the centrifuge tube and culture for a further 48 h. After further centrifugation, the lactate dehydrogenase activity detection kit (Solarbio, Beijing, China) was used to measure the activity of the supernatant.

### 5.4. HE Staining

Attached the cell culture coverslip to the bottom of the 24-well plate. Slowly dropped the cell suspension of 500 μL to the cell crawler, and placed it in CO_2_ incubator to wait for cell density cultured to 50–60% or so. Cultivated with FB_1_ solution (0, 10, 20, 40 μg/mL) for 48 h. The coverslip was taken for fixation, hematoxylin staining, differentiation, eosin staining, dehydration, transparency, mounting. The changes in nucleus and cytostyte are observed under a microscope.

### 5.5. Flow Cytometric Analysis

After 24 h of treatment with FB_1_ (0, 10, 20, 40 μg/mL), cells were suspended with ice-cold phosphate buffered saline (PBS) after centrifugation. 500 μL propidium iodide solution (Genview, Beijing, China) was added and the sample was incubated in the dark for 30 min. The cell cycle was detected with a flow cytometer. The cell proliferation index was calculated according to the data obtained from the flow cytometer test: Cell proliferation index (%) = ((G2/M + S)/(G0/G1 + S + G2/M)) ∗ 100%.

### 5.6. qRT-PCR Assay

The total RNA of SUVECs was extracted using the TransZol Up reagent (TransGen Biotech, Beijing, China) and cDNA was synthesized using the one-step reverse transcription kit (TransGen Biotech). Real-time quantitative polymerase chain reaction (qRT-PCR) was performed using the Bio-Rad Laboratories CFX Connect™ Real-Time PCR Detection System and calculated by the 2^−^^ΔΔct^ method. The primer sequences were constructed by Sangon Biotech (Shanghai) Co., Ltd., and are shown in Table 1. Glyceraldehyde-3-phosphate dehydrogenase (GADPH) were used as reference gene.

### 5.7. Western Blot

Cells were rinsed twice in ice-cold PBS. The RIPA buffer (Solarbio), protease inhibitor mixture (Solarbio), and protein phosphatase inhibitor (Solarbio) were subsequently added to the extract protein. Supernatants were used to quantify the protein concentration via the Bradford protein kit (Tiandz, Beijing, China). Proteins were separated by sodium dodecyl sulfate—polyacrylamide gel electrophoresis (SDS-PAGE) at 80 V for 40 min and 120 V for 50 min, and subsequently electrotransferred to polyvinylidene fluoride (PVDF) membranes at 200 mA for 80 min. Membranes were incubated with primary antibody P21 (Proteintech, Wuhan, China), P53 (Bioss, Beijing, China), PCNA (Bioss), p-Cyclin B1 (Bioss), Claudin1 (Bioss), Occludin (Bioss), ZO-1 (Bioss), Cdc25c (CST, Boston, MA, USA), and GAPDH (Servicebio, Wuhan, China) overnight at 4 °C. After incubation with corresponding secondary antibodies, the protein band densities were quantified using Bio-Rad ChemiDoc™ XRS and Image Lab™ Software.

### 5.8. Scanning Electron Microscopy (SEM)

FB_1_-treated sterilized coverslips were rinsed with PBS and the samples fixed in 2 mL 2.5% glutaraldehyde for 2 h. Next, samples were fixed for 1–2 h at room temperature with PBS, which included 1% osmium tetroxide. Afterwards, the coverslips were dehydrated in a series of graded ethanol concentrations, mounted with double-sided tape, and platinum-coated in a sputter coater for about 30 s. For visualization, the electron micrographs were taken and analyzed under a scanning electron microscope.

### 5.9. Transmission Electron Microscopy (TEM)

FB_1_-treated cells were rinsed with PBS and fixed in 2 mL 2.5% glutaraldehyde for 2 h at 4 °C. The fixed cells were scraped and pelleted in agarose. Next, the samples were fixed for 1–2 h at 20 °C with PBS, which included 1% osmium tetroxide. Afterwards, the samples were dehydrated in a series of graded ethanol concentrations. Cells were embedded using 812 embedding resin, and stained en bloc with uranyl acetate and lead citrate. Samples were viewed with a transmission electron microscope, and images were captured for analysis.

### 5.10. Paracellular Permeability Testing

SUVECs with DMEM were seeded in the upper chambers of transwell inserts. The lower chambers contained 600 μL DMEM with 10% FBS. After the cells were cultured, the fluids in the upper and lower chambers were replaced with FB_1_ (0, 10, 20, 40 μg/mL), and treated for 24, 48, and 72 h. Fluorescein isothiocyanate (FITC)-dextran (5 mg/mL) was added in the upper chamber and 600 μL PBS was added in the lower chamber. The lower chamber liquid was collected and the absorbance was measured. Paracellular permeability was calculated as follows: Paracellular permeability = FITC-dextran concentration in the lower chamber/the average FITC-dextran concentration of the control group.

### 5.11. Phalloidin Staining

SUVECs were treated with FB_1_ (0, 10, 20, 40 μg/mL) for 24 h and fixed in 4% polyformaldehyde for 30 min. The 50–100 μL permeabilization wash buffer was added, and samples were incubated for 10 min at room temperature followed by incubation with 50–100 μL phalloidin (Servicebio) for another 2 h. After incubation with 4′,6-diamidino-2-phenylindole (DAPI) stain solution (Servicebio) for 10 min, antifade mounting medium (Servicebio) was prepared to mount the coverslips. Finally, the cell culture coverslips were placed under an inverted fluorescence microscope to observe and capture the images.

### 5.12. Statistical Analysis

All data were expressed as mean ± standard deviation (SD) and resolved by one-way analysis of variance and independent sample *t*-test to analyze the differences between groups using Graphpad Prism 6.01. All experiments were repeated at least three times. *p* < 0.05 was considered significant (data marked with the *).

## Figures and Tables

**Table 1:** Primer sequences of genes for qRT-PCR.

Genes	Primer Sequences	Genebank No
p21	F: 5′-ACCCCTTCCCCATACCC-3′ R: 5′-TTCCTAACACCCATGAAACTG-3′	XM_013977858.2
p27	F:5′-GTCCCTTTCAGTGAGAACCG ATAC-3′ R: 5′-TTGCTGCCACATAACGGAATCAT-3′	NM_214316.1
p53	F: 5′-CTGCTTCCTGAAAACAACC-3′ R: 5′-AAGGGACAAAGGACGACA-3′	NM_213824.3
PCNA	F: 5′-GTGATTCCACCACCATGTTC-3′ R: 5′-TGAGACGACTCCATGCTCTG-3′	NM_001291925.1
Cyclin B1	F: 5′-AACTGCTCTTGGAGACATCGGT-3′ R: 5′-TGGTTCAGGCTCCAGTTCAGG-3′	NM_001170768.1
Cyclin D1	F: 5′-GCGAGGAACAGAAGTGCG-3′ R: 5′-TGGAGTTGTCGGTGTAGATGC-3′	XM_021082686.1
Cyclin E1	F: 5′-CTCGCCACTGCCTATACTGA-3′ R: 5′-GGTGCCGCTGCATAAGGT-3′	XM_005653265.2
CDK-4	F: 5′-GCGGAGATTGGTGTTGGTG-3′ R: 5′-CATTGGGGACTCTTACGCTCTT-3′	NM_001123097.1
Claudin 1	F: 5′- GCAGCAGCTTCTTGCTTCTC-3′ R: 5′-CTGGCATTGACTGGGGTCAT-3′	NM_001244539.1
Occludin	F: 5′-ATCAACAAAGGCAACTCT -3′ R: 5′-GCAGCAGCCATGTACTCT -3′	XM_005672525.3
ZO-1	F: 5′-GAGTTTGATATGGGCGTT -3′ R: 5′-GTGGGAGGATGCTGTTGT -3′	XM_021098896.1
GAPDH	F: 5′-ACAGGGTGGTGGACCTCATG -3′ R: 5′-GGGTCTGGGATGGAAACTGG -3′	XM_021091114.1

## Data Availability

Not applicable.
